# CAIX Regulates GBM Motility and TAM Adhesion and Polarization through EGFR/STAT3 under Hypoxic Conditions

**DOI:** 10.3390/ijms21165838

**Published:** 2020-08-14

**Authors:** Bor-Ren Huang, Yu-Shu Liu, Sheng-Wei Lai, Hui-Jung Lin, Ching-Kai Shen, Liang-Yo Yang, Dah-Yuu Lu

**Affiliations:** 1 Department of Neurosurgery, Taichung Tzu Chi Hospital, Buddhist Tzu Chi Medical Foundation, Taichung 42743, Taiwan; smallblue525@hotmail.com; 2 School of Medicine, Tzu Chi University, Hualien 97004, Taiwan; 3 Department of Pharmacology, School of Medicine, China Medical University, Taichung 40402, Taiwan; yushuliu220@gmail.com (Y.-S.L.); linzoe9260@gmail.com (H.-J.L.); 4 Department of Physiology, School of Medicine, China Medical University, Taichung 40402, Taiwan; 5 Graduate Institute of Basic Medical Science, China Medical University, Taichung 40402, Taiwan; wayson081024@gmail.com; 6 Graduate Institute of Biomedical Sciences, China Medical University, Taichung 40402, Taiwan; b0953295012@gmail.com; 7 Laboratory for Neural Repair, China Medical University Hospital, Taichung 40402, Taiwan; 8 Biomedical Technology R&D Center, China Medical University Hospital, Taichung 40402, Taiwan; 9 Department of Photonics and Communication Engineering, Asia University, Taichung 41354, Taiwan

**Keywords:** CAIX (carbonic anhydrase IX), hypoxic condition, GBM (glioblastoma multiforme), motility, M2 polarization

## Abstract

Carbonic anhydrases (CAs) are acid–base regulatory proteins that modulate a variety of physiological functions. Recent findings have shown that CAIX is particularly upregulated in glioblastoma multiforme (GBM) and is associated with a poor patient outcome and survival rate. An analysis of the GSE4290 dataset of patients with gliomas showed that CAIX was highly expressed in GBM and was negatively associated with prognosis. The expression of CAIX under hypoxic conditions in GBM significantly increased in protein, mRNA, and transcriptional activity. Importantly, CAIX upregulation also regulated GBM motility, monocyte adhesion to GBM, and the polarization of tumor-associated monocytes/macrophages (TAM). Furthermore, the overexpression of CAIX was observed in intracranial GBM cells. Additionally, epidermal growth factor receptor/signal transducer and activator of transcription 3 regulated CAIX expression under hypoxic conditions by affecting the stability of hypoxia-inducible factor 1α. In contrast, the knockdown of CAIX dramatically abrogated the change in GBM motility and monocyte adhesion to GBM under hypoxic conditions. Our results provide a comprehensive understanding of the mechanisms of CAIX in the GBM microenvironment. Hence, novel therapeutic targets of GBM progression are possibly developed.

## 1. Introduction

Glioblastoma multiforme (GBM) is considered one of the most deadly brain tumors because of its aggressive invasive growth and resistance to chemotherapy and radiotherapy [1,2]. A histological analysis of GBM revealed a heterogeneous cellular composition of neoplastic glioma cells and nonneoplastic cells that form a tumor microenvironment [3]. Recently, increasing evidence has suggested that nonneoplastic cells in the tumor microenvironment are important for the maintenance of cancer growth and response to therapy. The most abundant populations of tumor-associated macrophages (TAMs) in the glioma microenvironment include resident brain microglia, infiltrating blood monocytes, and macrophages from the blood circulation [3]. As far TAMs have been studied, more M2-type (anti-inflammatory) TAMs are observed in tumor sites, and the number of M2-type TAMs increases as the tumor progresses [4]. Specific histological characteristics including pseudopalisading necrosis and microvascular proliferation differentiate GBM from lower-grade gliomas, and these unique hallmarks make GBM one of the most hypoxic tumors [5,6]. These specific niches within the tumor microenvironment may change metabolic sources, the acid–base microenvironment, and immune surveillance [7,8,9]. A recent report also indicated that decreasing the tumor-promoting effects of macrophages in GBM can be considered a potential strategy in combination with standard glioma therapies [10].

Oncogenic metabolism, which comprises a variable proportion of glycolysis, leads to the excessive production of acidic metabolic products, including lactate and CO_2_ [11]. Intratumoral oxygenation leads to hypoxia, acidosis, and necrosis, and it is a major cause of morbidity in GBM patients [12]. Hypoxia-induced pH-regulating proteins cause the acidosis of tumor microenvironments, which may act as a critical mediator of tumor progression and the tumor escape of immune surveillance [13,14,15,16,17]. For instance, tumors release acidic metabolite lactate, which induces the expression of vascular endothelial growth factor (VEGF) and the M2 polarization of TAMs [16] subsequently promoting tumor growth. Furthermore, neutralizing intratumoral acidity reduces the pro-tumor phenotype of macrophages while also decreasing tumor incidence and invasion [17]. In clinical observation, acidosis is associated with radio- or chemotherapy resistance [18,19], suppressed immunity, and cytotoxic T-cell responses [20]. In a mouse model, neutralizing tumor acidosis was found to impair the growth of some cancer types that are associated with increased cytotoxic T-cell infiltration [21]. Cancer is considered a cellular metabolic disease [22] that links oncogenesis with the pentose cycle and fat metabolism [23] for drug development [24]. A variety of hypoxia-induced proteins that promote cellular anabolism and the regulation of metabolic acid–base levels in tumor cells including monocarboxylate transporters (MCTs), carbonic anhydrases (CAs), and bicarbonate transporters (NBCs) [25]. Our recent findings showed that MCT4 is increased in the tumor necrotic tissues, and MCT4 promotes GBM cell motility and monocyte adhesion to GBM cells [26].

CAs are acid–base regulatory proteins that modulate various physiological processes, such as acid–base balance, respiration, carbon dioxide and ion transport, and bone resorption [27]. CAs catalyze a physiological reaction, and the conversion of carbon dioxide (CO_2_) and water into bicarbonate (HCO_3_^−^) and protons (H^+^) and HCO_3_^−^ transported back to cells produces extracellular H^+^ and intracellular HCO_3_^−^, thereby participating in intracellular alkalinization and extracellular acidification [28]. Previous studies have reported that CAIX might provide tumor cells with a survival advantage under hypoxic conditions or acidosis [29,30]. On the other hand, naturally occurring deuterium is essential for normal cell growth, and deuterium-depleted water (DDW) regulates mitochondrial functions, cell growth, and oxygen delivery [31]. DDW is considered to be associated with cancer prevention and treatment using natural ketogenic diets and low deuterium drinking water [32]. CAIX inhibitors have been reported to decrease the uptake of extracellular deuterated water into cells in childhood brain tumor neuroblastoma [32]. Moreover, CAIX has been considered a prognostic and therapeutic biomarker in medulloblastomas and supratentorial primitive neuroectodermal tumors [33]. A recent study indicated that CAIX has a high expression in pre-cancerous lesions in pancreatic cancer [34]. Importantly, the application of CAIX inhibitors with improved potency can significantly affect pancreatic cancer cell survival [35]. CAIX is a poor prognostic marker in a large cohort of breast cancer patients, and targeting CAIX activity with selective small molecule inhibitors of CAIX attenuates the growth of breast tumors [30]. Furthermore, SLC-0111, a high selective CAIX inhibitor, improves the efficacy of temozolomide to extend the survival of GBM-bearing mice [36]. Currently, an ongoing clinical trial (NCT03450018) is using the selective inhibitor SLC-0111 in CAIX-positive patients diagnosed with metastatic pancreatic ductal adenocarcinoma [34].

Molecular mechanism responses to hypoxia are predominantly mediated by the hypoxia-inducible factor (HIF)-1α [37], which transactivates several genes essential for adaptation and is maintains tumor survival [38]. HIF-1α is also considered to be a key molecule in the transactivation of genes implicated in altered metabolism, leading to the accumulation of several types of metabolites in the microenvironment that promote tumor growth and aggressiveness [39,40]. CAIX expression is associated with malignancy in gliomas [41], and CAIX elevation in GBM is also associated with poor prognosis [29]. CAIX is recognized as the downstream of HIF-1α. Importantly, HIF-1α is also overexpressed in human glioblastoma specimens and is associated with poor patient survival [42]. Phosphoinositide 3 kinase and mitogen-activated protein (MAP) kinase signaling are involved in the regulation of CAIX expression under hypoxic conditions [43]. The MAP kinase upregulates HIF-1α activity through the CREB-binding protein/p300 coactivator [44]. Recently, we also found that the p300 cofactor is involved in signal transducer and activator of transcription 3 (STAT3)-mediated interleukin (IL)-8 production in GBM [45]. Additionally, the recruitment of the p300 coactivator is associated with HIF-1α nuclear translocation in hypoxic cells [46]. Moreover, the recruitment of STAT3, HIF-1α, and p300 under hypoxic conditions induces histone H3 acetylation and VEGF gene expression in human renal carcinoma cells [47]. Moreover, the tumor-immunosuppressed programmed death ligand 1 (PD-L1) is expressed via HIF-1α and STAT3 under hypoxic conditions in lung cancer [48]. Importantly, the activation of the STAT3/HIF-1α axis promotes trastuzumab resistance in HER2-positive breast cancer cells [49]. In genomic analysis, the presence of abnormalities in the epidermal growth factor receptor (EGFR) expression of glioblastoma is an important characteristic with clinically relevant subtypes [50]. Our previous finding suggested that EGFR activation is associated with the activation of STAT3 signaling in the stimulation of an inflammatory response in human nucleus pulposus cells [51]. Furthermore, we also found that EGFR-dependent adhesion molecule expression and monocyte adhesion to GBM is mediated by the STAT3 signaling pathway [52]. Our recent report also showed that hypoxia mediated the acetylated STAT3 expression and regulated the transcriptional activity of HIF-1α in GBM [26]. The present study aimed to determine the modulation of CAIX in GBM motility, as well as the adhesion and polarization of monocytes under hypoxic conditions. Additionally, we investigated the molecular regulation of EGFR and STAT3 in HIF-1α and CAIX expression.

## 2. Results

### 2.1. Expression of CAIX and pH-Regulating Proteins through Hypoxia-Inducible Factor 1α (HIF-1α) Activation under Hypoxic Conditions

**Figure 1:**
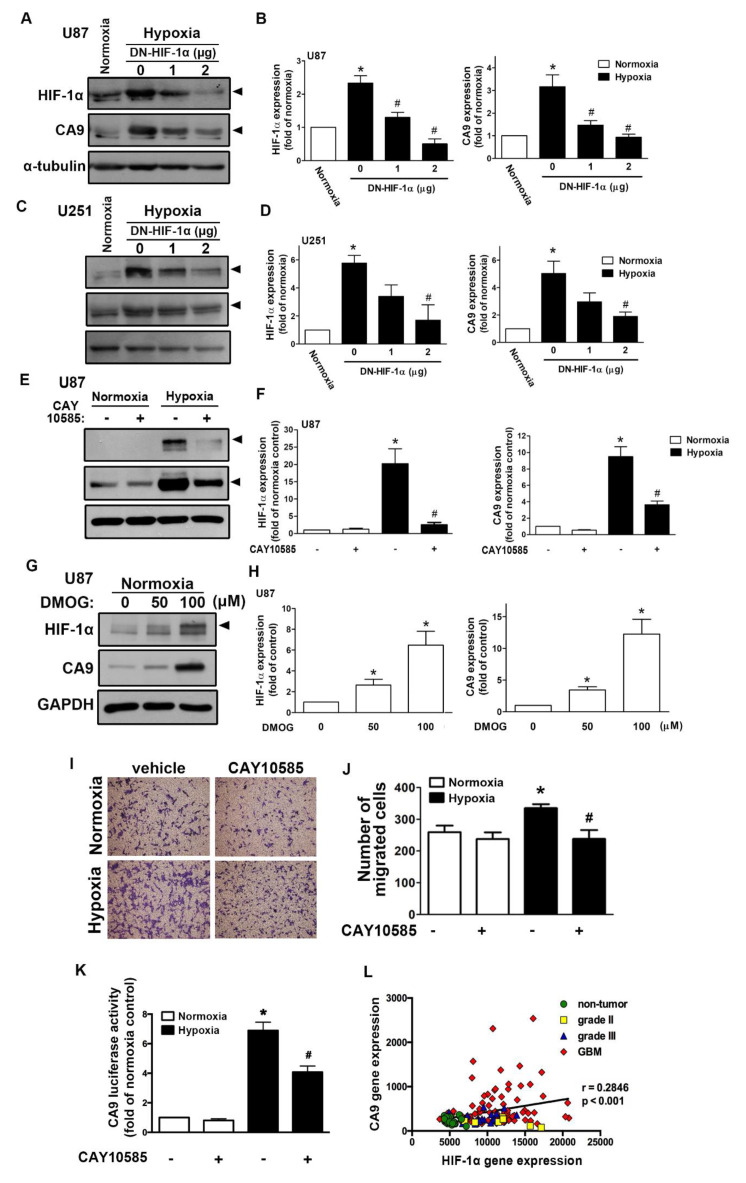
Hypoxia-inducible factor (HIF)-1α is involved in the hypoxia-induced CAIX expression and glioblastoma multiforme (GBM) migration. U87 (**A**) and U251 (**C**) were transfected with a dominant-negative mutant of HIF-1α (1 or 2 μg) for 24 h and exposed to hypoxia (1% oxygen (O_2_)) for another 24 h. The quantitative results are shown in a bar graph (**B**,**D**). (**E**) U87 was treated with an HIF-1α inhibitor (10 μM) for 30 min and exposed to hypoxic conditions (1% O_2_) for another 24 h. The quantitative results are shown in a bar graph (**F**). (**G**) U87 was treated with dimethyloxalylglycine (50 or 100 μM) for 24 h. HIF-1α and CAIX protein expression were determined by Western blotting using whole cell lysates. The quantitative results are shown in a bar graph (**H**). (**I**) U87 cells were treated with an HIF-1α inhibitor (10 μM) for 30 min and exposed to hypoxic conditions (1% O_2_), and in vitro migration activities were measured after 24 h with a transwell assay and then visualized using a digital camera. (**J**) The quantification of U87 migration by the number of cells that migrated to the underside of the filter. (**K**) U87 cells were transfected with CAIX luciferase reporter plasmids for 24 h, treated with an HIF-1α inhibitor (10 μM) for 30 min, and exposed to hypoxic conditions (1% O_2_). Firefly luciferase activities were measured after 24 h, and the results were normalized to the renilla luciferase activity. * *p* < 0.05 compared with the normoxia control group. # *p* < 0.05 compared with the hypoxia group. A two-way analysis of variance with a post-hoc Bonferroni test was used to examine the significance of the mean. Quantitative data are presented as the mean ± standard error (representative of *n* = 3). (**L**) Pearson’s correlation analysis between HIF-1α and CAIX expression in human glioma microarray dataset GSE4290 (*r* = 0.2846, *p* < 0.001).

We first assessed the hypoxia-mediated pH-regulating protein expression in GBM by qPCR. As shown in Figure S1A, hypoxia induced CAIX, monocarboxylate transporter 4, sodium–hydrogen exchanger 1 (NHE1), glucose transporter (GLUT)1, and GLUT3 expression. Additionally, CAIX levels in GBM were also clearly higher under long-term hypoxic conditions (Figure S1B). Moreover, analysis of the GSE4290 dataset showed that levels of CAIX were higher in the GBM group than those in the astrocytoma (grades II and III) and non-tumor groups (Figure S1C). However, NHE1 expression did not significantly differ between the astrocytoma group (from grade II to GBM) and the non-tumor group (Figure S1D). We further analyzed the gene dataset of patients with glioma and showed that the levels of HIF-1α were higher in the astrocytoma group (from grade II to GBM) than those in the non-tumor group (Figure S1E). Similarly, hypoxia induced HIF-1α and CAIX protein expression in a time-dependent manner in both human and mouse GBMs (Figure S2A–C). Moreover, hypoxia also induced CAIX mRNA expression in a time-dependent manner (Figure S2E,F). Interestingly, hypoxia markedly induced HIF-1α protein expression (Figure S2B–D), but it did not affect HIF-1α mRNA expression (Figure S2D,E). We subsequently examined whether HIF-1α modulated CAIX expression under hypoxic conditions. Transfection with an HIF-1α dominant-negative (DN) mutant reduced the hypoxia-induced HIF-1α expression and CAIX expression in two different human GBMs (Figure 1A–D). Additionally, treatment with an HIF-1α inhibitor also decreased hypoxia-induced CAIX expression (Figure 1E,F). In contrast, treatment with an HIF-hydroxylase inhibitor—dimethyloxalylglycine (DMOG)—stabilized the HIF-1α protein, which increased CAIX expression (Figure 1G,H). Furthermore, human GBM cells treated with an HIF-1α inhibitor antagonized hypoxia-enhanced GBM migration (Figure 1I,J).

Additionally, GBM was exposed to hypoxia-induced CAIX transcriptional activity, and GBM cells treated with an HIF-1α inhibitor reversed the hypoxia-enhanced CAIX expression (Figure 1K). Finally, Pearson’s correlation analysis also showed a positive association between HIF-1α and CAIX expression levels in the gene expression dataset of patients with glioma (Figure 1L). These results suggest that hypoxia stimulates HIF-1α protein stabilization and CAIX expression in GBM, which enhances GBM motility. In GBM patients, higher levels of HIF-1α are positively associated with CAIX expression in GBM specimens.

### 2.2. Enhancement of CAIX Modulates Glioblastoma Multiforme (GBM) Motility under Hypoxic Conditions

**Figure 2:**
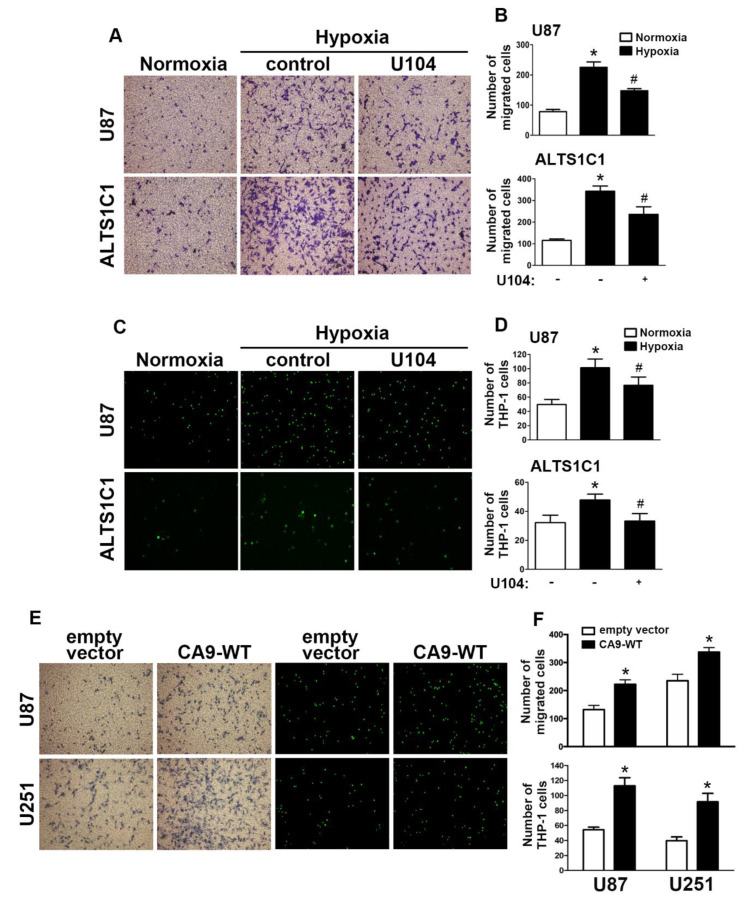
CAIX is involved in the hypoxia-enhanced GBM migration and monocyte adhesion. (**A**) U87 and ALTS1C1 were treated with a CAIX inhibitor (U104; 3 μM) for 30 min and exposed to hypoxic conditions (1% oxygen (O_2_)), and in vitro migration activities were measured after 24 h with a transwell assay and then visualized using a digital camera. (**B**) The quantification of U87 (upper panel) and ALTS1C1 (lower panel) migration by the number of cells that migrated to the underside of the filter. (**C**) U87 and ALTS1C1 were treated with a CAIX inhibitor (U104, 3 μM) for 30 min and exposed to hypoxic conditions (1% O_2_) for 24 h. BCECF-AM-labeled-THP-1 was added to U87 and ALTS1C1 for 30 min, and subsequently, the adherence of THP-1 was measured by fluorescence microscopy. (**D**) The quantification of monocyte adhesion abilities by THP-1 cell adhesion on U87 (upper panel) or ALTS1C1 (lower panel). * *p* < 0.05 compared with the normoxia group. # *p* < 0.05 compared with the hypoxia group. A two-way analysis of variance with a post-hoc Bonferroni test was used to examine the significance of the mean. (**E**) U87 and U251 cells were transfected with empty vector or wild-type CAIX for 24 h and exposed to hypoxia (1% O_2_) for 24 h. In vitro migration activities (left panel) and monocyte adhesion abilities (right panel) were measured using a digital camera and fluorescence microscopy, respectively. (**F**) The quantification of GBM migration abilities (upper panel) and monocyte adhesion abilities (lower panel). * *p* < 0.05 compared with the empty vector group (Student’s *t*-test). Quantitative data are presented as the mean ± standard error (representative of *n* = 3).

Next, we aimed to further investigate CAIX in the modulation of hypoxia stimulated in GBM. A transwell assay was performed to examine whether CAIX facilitated GBM migration under hypoxic conditions. Microscopic images (Figure 2A) indicated that treatment with U104 (a CAIX inhibitor) resulted in decreased hypoxia-enhanced GBM migration activity in both U87 and ALTS1C1 (Figure 2B). Additionally, CAIX mediated the binding of monocytes to GBM and was further determined using the monocyte-binding assay. As shown in Figure 2C,D, the treatment of GBMs with U104 decreased hypoxia-enhanced monocyte adhesion (the green color in Figure 2C). Moreover, transfection with wild-type CAIX increased CAIX protein expression (Figure S3) and subsequently enhanced GBM migration activity and monocyte adhesion in both U87 and U251 cells (Figure 2E,F).

**Figure 3:**
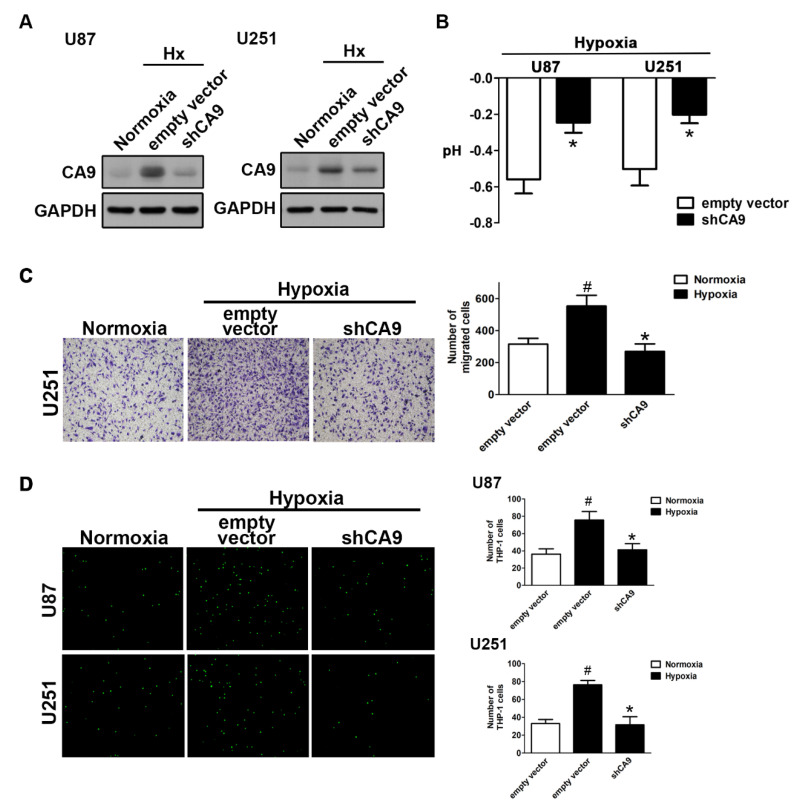
The knockdown of CAIX reduces hypoxia-induced GBM motility and monocyte adhesion to GBM. (**A**) U87 and U251 cells were transfected with empty vector or wild-type CAIX for 24 h and exposed to hypoxic conditions (1% O_2_) for 24 h. CAIX expressions were determined by Western blot using whole cell lysates. The pH values of the culture media are shown in (**B**). In vitro migration activities were measured after 24 h with a transwell assay and then visualized using a digital camera (**C**, left panel). The quantification of GBM migration abilities is shown in the right panel. BCECF-AM-labeled-THP-1 was added to GBM cells for 30 min, and, subsequently, the adherence of THP-1 was measured by fluorescence microscopy (**D**, left panel). The monocyte adhesion abilities are shown in right panel. * *p* < 0.05 compared with the empty vector group. # *p* < 0.05 compared with the shCAIX group. (Student’s *t*-test). Quantitative data are presented as the mean ± standard error (representative of *n* = 3).

As expected, the knockdown of CAIX by transfection with CAIX shRNA under hypoxic conditions reduced the enhancement of CAIX expression (Figure 3A) and attenuated the reduction of the pH value (Figure 3B). Moreover, both the enhancement of GBM motility (Figure 3C) and monocyte adhesion (Figure 3D) under hypoxic conditions were reduced by the knockdown of CAIX in GBM. These results indicated that hypoxia-induced CAIX expression markedly enhances GBM migration and GBM-associated monocyte adhesion.

### 2.3. The GBM Microenvironment Regulates Monocyte Polarization under Hypoxic Conditions

**Figure 4:**
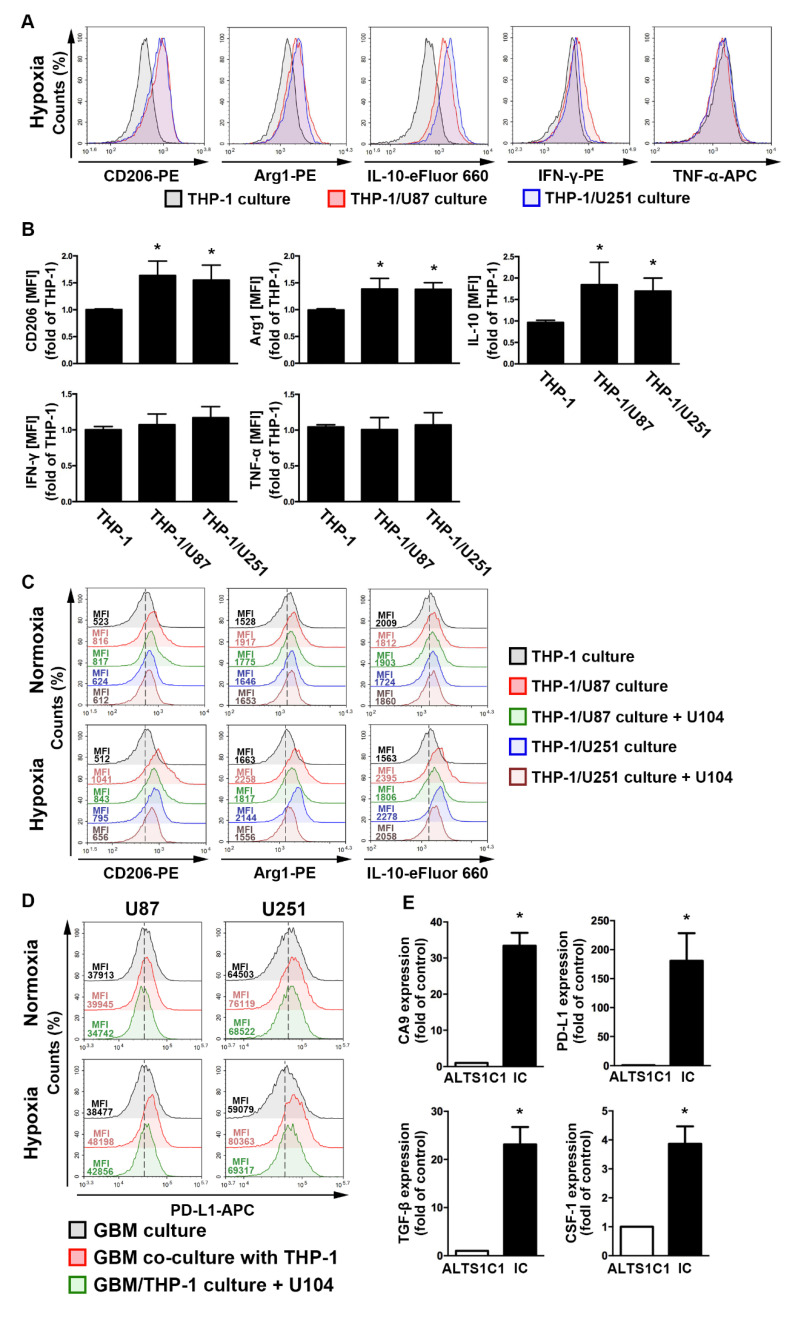
The polarizations of monocyte and GBM progression in a co-cultured model under hypoxic conditions. (**A**) THP-1 monocytes were co-cultured with U87-green fluorescent protein (GFP) or U251-GFP under hypoxic conditions for 48 h. The co-cultured cells were plotted on a side scatter versus FITC. The THP-1 monocytes (GFP-negative cells) were analyzed to assess the levels of cluster of differentiation (CD) 206, arginase 1 (Arg1), interleukin (IL)-10, interferon (IFN)-γ, and tumor necrosis factor (TNF)-α by flow cytometry. (**B**) The median fluorescence intensity (MFI) of CD206, Arg1, IL-10, IFN-γ, and TNF-α in THP-1 monocytes in the co-cultured model under hypoxic conditions for 48 h. * *p* < 0.05 compared with the THP-1 monocyte group. Quantitative data are presented as the mean ± standard error (representative of *n* = 3). (**C**) U87-GFP and U251-GFP were treated with U104 (CAIX inhibitor) and subsequently co-cultured with THP-1 monocytes under hypoxic conditions for 48 h. THP-1 monocytes (GFP-negative cells) were analyzed to assess the levels of CD206, Arg1, and IL-10 by flow cytometry. (**D**) U87-GFP or U251-GFP was treated with U104 and subsequently co-cultured with THP-1 monocytes under hypoxic conditions for 48 h. The co-cultured cells were plotted on a side scatter versus FITC. The GFP-positive gated GBM was analyzed to assess the levels of programmed death ligand 1 (PD-L1) by flow cytometry. (**E**) The levels of CAIX, PD-L1, tumor growth factor-β, and colony-stimulating factor mRNA in intracranial GBM of ALTS1C1-bearing mice were examined by quantitative polymerase chain reaction on day 24. * *p* < 0.05 compared with the naive control (ALTS1C1 GBM) (Student’s *t*-test). Quantitative data are presented as the mean ± standard error (representative of *n* = 4).

To assess the regulation of the acidic microenvironment in immunity, monocyte and GBM co-culture assays were performed. In the GBM-monocyte co-cultured system, THP-1 monocytes significantly increased the levels of M2 markers, including the cluster of differentiation (CD)206, arginase 1 (Arg1), and interleukin (IL)-10, under hypoxic conditions (Figure 4A,B). Interestingly, the expression of the M1 markers interferon (IFN)-γ and tumor necrosis factor (TNF)-α did not change in the co-cultured THP-1 monocytes (Figure 4A,B). Importantly, co-cultured cells treated with the CAIX inhibitor U104 dramatically attenuated the hypoxia-induced expression of M2 markers on THP-1 monocytes (Figure 4C). Moreover, the co-cultured GBM exhibited higher levels of PD-L1 than the mono-cultured model (Figure 4D). Furthermore, co-cultured cells treated with U104 effectively reduced the hypoxia-induced PD-L1 expression on the GBM surface (Figure 4D). In addition, there was no statistically significant difference between the hypoxic and normoxic conditions in the PD-L1 expression on THP-1 monocytes (Figure S4). Furthermore, an analysis of the mouse GBM of ALTS1C1-bearing mice showed that levels of CAIX, PD-L1, transforming growth factor-β, and colony-stimulating factor 1 were significantly higher in the intracranial GBM than those in the ALTS1C1 GBM group (Figure 4E). These results suggest that CAIX regulates monocyte M2 polarization in a hypoxic GBM microenvironment that expresses immunosuppressive molecules on GBM, which may lead to tumor progression.

### 2.4. Regulation of Epidermal Growth Factor Receptor/Signal Transducer and Activator of Transcription 3 in Hypoxia-Induced HIF-1α and CAIX Expression in GBM

**Figure 5:**
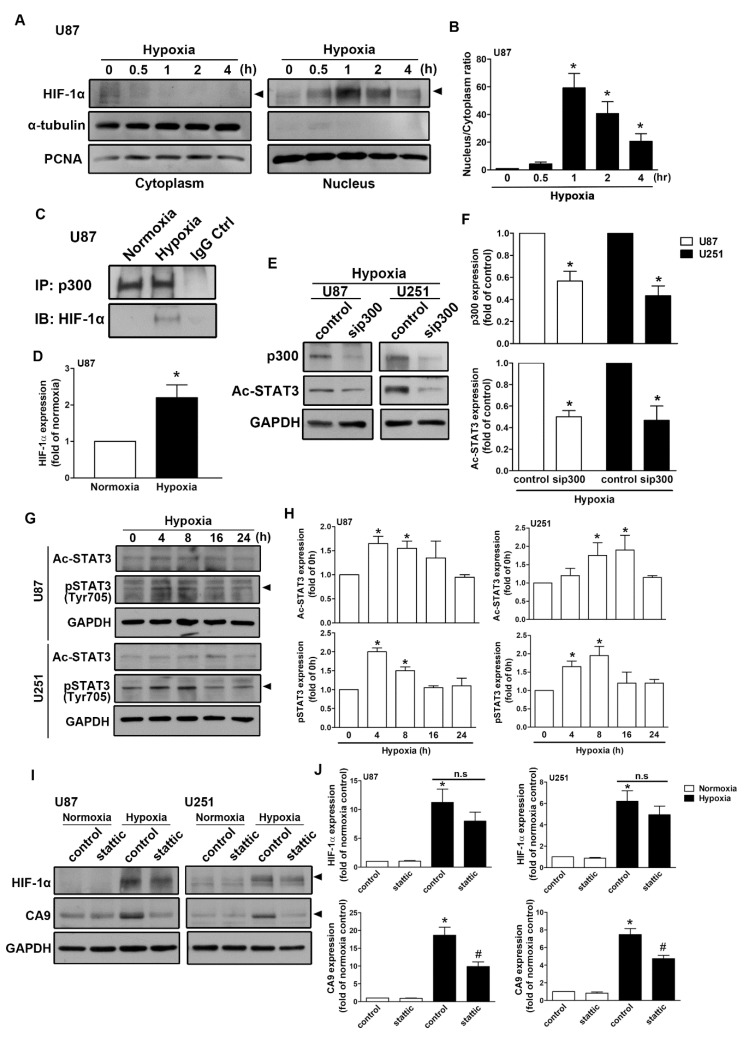
Signal transducer and activator of transcription 3 (STAT3) is involved in hypoxia-induced HIF-1α and CAIX expression in GBM. (**A**) The cytosolic and nuclear extracts from hypoxia-incubated (1% oxygen (O_2_) for 0.5, 1, 2, or 4 h) U87 cells were subjected to Western blotting. The expression levels of HIF-1α were determined using Western blotting. The quantitative results are shown in a bar graph (**B**). (**C**) U87 cells were exposed to hypoxic conditions (1% O_2_) for 4 h, and co-immunoprecipitation experiments were performed by precipitating the whole cell lysate (1 mg) with p300 and nonspecific immunoglobulin G antibodies using protein G magnetic beads. HIF-1α and p300 expression levels were determined using Western blot analysis. The quantitative results are shown in a bar graph (**D**). (**E**) U87 and U251 were transfected with control or p300 small interfering RNA for 24 h and subsequently exposed to hypoxic conditions (1% O_2_) for another 24 h. Acetyl-STAT3 and p300 expressions were determined by Western blot using whole cell lysates. The quantitative results are shown in a bar graph (**F**). (**G**) U87 and U251 were exposed to hypoxic conditions (1% O_2_) for the indicated time periods (4, 8, 16, or 24 h), and acetyl-STAT3 and phospho-STAT3 expressions were determined by Western blot using whole cell lysates. The quantitative results are shown in a bar graph (**H**). (**I**) U87 and U251 were treated with a STAT3 inhibitor (10 μM) for 30 min and exposed to hypoxic conditions (1% O_2_) for 24 h. HIF-1α and CAIX expressions were determined by Western blot using whole cell lysates. The quantitative results are shown in a bar graph (**J**). * *p* < 0.05 compared with the normoxic control group. # *p* < 0.05 compared with the hypoxic group. (Student’s *t*-test).

**Figure 6:**
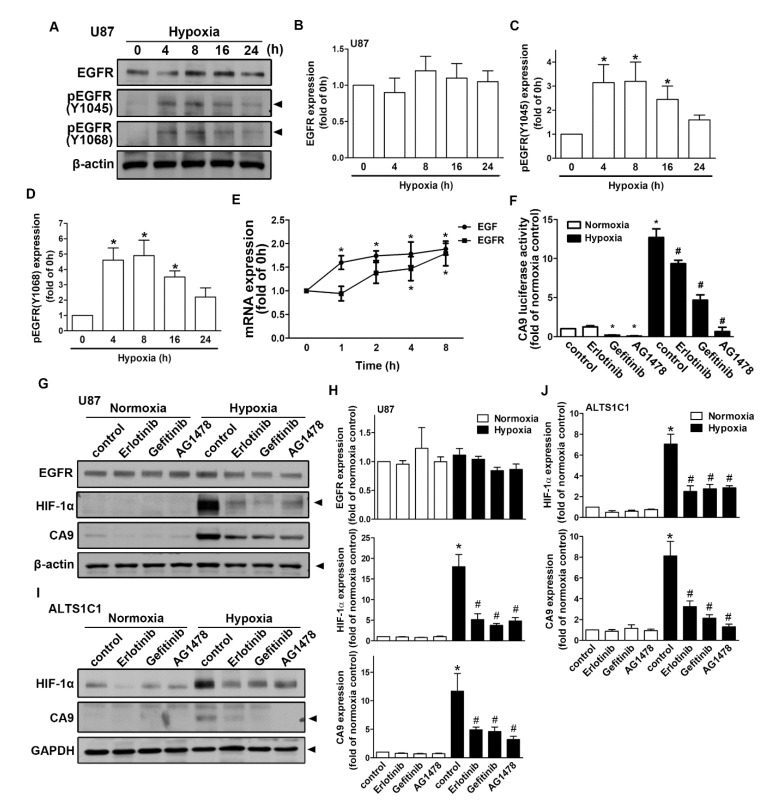
The activation of epidermal growth factor receptor (EGFR) is involved in the hypoxia-induced HIF-1α and CAIX expression in GBM. (**A**) U87 cells were exposed to hypoxic conditions (1% oxygen (O_2_)) for the indicated time periods (4, 8, 16, or 24 h), and phosphorylated EGFR (Tyr1045 and Tyr1068) and EGFR expressions were determined by Western blot using whole cell lysates. The quantitative results are shown in a bar graph (**B**–**D**). (**E**) U87 cells were exposed to hypoxic conditions (1% O_2_) for the indicated time periods (1, 2, 4, or 8 h), and the mRNA expressions of EGF and EGFR were determined using qPCR. (**F**) U87 cells were transfected with CAIX luciferase reporter plasmids for 24 h and treated with EGFR inhibitors (50 nM erlotinib, 50 nM gefitinib, and 10 μM AG1478) for 30 min and then subsequently exposed to hypoxic conditions (1% O_2_) for another 24 h. Firefly luciferase activities were measured, and the results were normalized to the renilla luciferase activities. U87 (**G**) and ALTS1C1 (**I**) were treated with EGFR inhibitors (50 nM erlotinib, 50 nM gefitinib, and 10 μM AG1478) for 30 min and exposed to hypoxic conditions (1% O_2_) for another 24 h. EGFR, HIF-1α, and CAIX expressions were determined by Western blot using whole cell lysates. The quantitative results are shown in a bar graph (**H**,**J**). * *p* < 0.05 compared with the normoxia control group. # *p* < 0.05 compared with the hypoxia group. Quantitative data are presented as the mean ± standard error (representative of *n* = 3).

As shown in Figure 5A, human GBM exposed to hypoxia resulted in the translocation of HIF-1α from the cytoplasm to the nucleus. The nuclear translocation of HIF-1α peaked at 1 h and was sustained for 4 h (Figure 5B). Moreover, GBM exposed to hypoxia increased the interaction between HIF-1α and p300 acetyltransferase (Figure 5C,D). Additionally, transfection with p300 small interfering RNA (siRNA) decreased STAT3 acetylation in both human GBM U87 and U251 cells (Figure 5E,F). Additionally, GBM cells were incubated under hypoxic conditions, which increased STAT3 acetylation and phosphorylation in a time-dependent manner (Figure 5G,H). Importantly, the inhibition of STAT3 by stattic effectively antagonized hypoxia-induced CAIX expression (Figure 5I,J) in both U87 and U251 cells. Furthermore, the enhancement of HIF-1α was mildly decreased after the treatment of stattic (Figure 5I,J). As shown in Figure 6A–D, exposure to hypoxic conditions increased the levels of phospho-EGF receptor (Tyr1045 and Tyr1068) in a time-dependent manner. Additionally, we also found that GBM exposed to hypoxia increased EGF and EGFR mRNA expression in a time-dependent manner (Figure 6E). Subsequently, we investigated whether EGFR is involved in HIF-1α-mediated CAIX expression. Treatment with EGFR inhibitors significantly reduced hypoxia-enhanced CAIX transcriptional activity (Figure 6F). In addition, treatment with EGFR tyrosine kinase inhibitors, erlotinib, gefitinib, or AG1478, reduced hypoxia-induced HIF-1α and CAIX expression in human GBM (Figure 6G,H). Similarly, the inhibition of EGFR also decreased the enhancement of HIF-1α and CAIX expression in mouse GBM (Figure 6I,J). These results indicated that EGFR/STAT3 signaling is an important effector of the hypoxia-mediated stabilization of HIF-1α protein and CAIX expression in GBM.

## 3. Discussion

Developing effective treatment to GBM patients is urgently required with a better understanding of the molecular mechanisms of the GBM microenvironment in malignant progression to determine potential targets. With DNA microarray (Gene Expression Omnibus (GEO) dataset) analysis, we found that the higher expression of CAIX in patients with glioma was associated with poor prognosis. First, the present study demonstrated that CAIX and pH-regulating protein expressions were observed as a result of GBM response to hypoxia. The levels of CAIX showed the highest expression, and it was the most important among these pH-regulating proteins. Importantly, a positive association between CAIX expression and cell motility and adherent monocyte abilities in GBM was observed. Additionally, the overexpression of CAIX also increased GBM motility and monocyte adhesion under normoxic conditions. Second, the GBM-monocyte co-cultured system showed an increased M2 polarization in monocytes that also enhanced CAIX and immune checkpoint ligand PD-L1 expression in GBM. The intracranial GBM expressed significantly higher levels of CAIX, PD-L1, tumor growth factor (TGF)-β, and colony-stimulating factor (CSF)-1 than ALST1C1 GBM. Third, Pearson’s correlation analysis showed a positive association between the expressions of CAIX and HIF-1α in patients with glioma. Additionally, the upregulation of CAIX expression was dependent on HIF-1α protein stability, and an HIF-1α inhibitor or DN mutant effectively reduced CAIX expression and subsequently antagonized GBM motility. Fourth, we found that the GBM response to hypoxia resulted in the translocation of HIF-1α from the cytoplasm to the nucleus. Using co-immunoprecipitation (IP) assays, we also found that HIF-1α interacted with p300 acetyltransferase under hypoxic conditions, increasing STAT3 acetylation in a time-dependent manner. Moreover, the knockdown of p300 dramatically reduced the hypoxia-induced levels of Ac-STAT3. Furthermore, the inhibition of STAT3 effectively reduced hypoxia-induced CAIX expression. Finally, incubation under hypoxic conditions increased EGF and EGFR expressions, as well as phosphorylated EGFR levels, in a time-dependent manner. Importantly, the stability of the HIF-1α protein and the upregulation of CAIX expression were dependent on the activation of EGFR, and treatment with erlotinib, gefitinib, and AG1478 effectively reduced the enhancement of HIF-1α and CAIX expression, as well as CAIX transcriptional activities. These results suggest that hypoxia-induced CAIX expression is beneficial for GBM progression by increasing cell motility and monocyte adhesion. Subsequently, GBM interacts with monocytes and further increases M2-polarized macrophages and immune checkpoint ligand PD-L1 expression in GBM, which possibly changes the GBM microenvironment to escape immune surveillance.

Acidosis was found to significantly increase the formation of cancer metastases in vivo, and cellular motility was markedly higher at pH 6.6 than at pH 7.4 in lung cancer [53]. Acidosis decreased the gene expression of the pro-inflammatory markers Nos2, Ccl2, and IL-6 in IFN-γ/lipopolysaccharide-polarized macrophages, and it increased the expression of anti-inflammatory markers CD206 and Arg1 in IL-4-polarized macrophages [17]. CAIX plays a critical role in the expansion of breast cancer stem cells in hypoxic regions by sustaining the mesenchymal and stemness phenotypes [54]. In lung cancer patients, the expression level of CAIX is associated with tumor angiogenesis, apoptosis inhibition, and a loss of cell adhesion molecules, all of which explain the association between CAIX and poor outcomes [55]. Interestingly, the constitutive expression of the human CAIX protein modulates cell adhesion via interaction with β-catenin [56]. The CAIX protein directly localizes in focal adhesion structures and can promote initial adhesion through its proteoglycan domain [57]. Furthermore, synthetic oligopeptides compete for the M75 epitope of the proteoglycan domain, which inhibits the adhesion of cells to CAIX [58]. Importantly, CAIX expression is associated with malignancy in gliomas [41], and CAIX elevation in GBM is associated with a poor prognosis [29]. Moreover, CAIX expression in glioma cells is associated with poor response to classical anticancer therapeutic approaches such as chemo- and radiotherapy [59]. Importantly, used the small molecules against CAIX, as a combination therapy with temozolomide, improve the chemosensitivity of GBM and extend the survival of GBM-bearing mice [36,60]. It has been reported that hypoxia stimulates glycolytic metabolism, which generates increased amounts of lactic and carbonic acids and enhances the acidosis of the tumor microenvironment [61]. Under these conditions, GBM migration and invasion were found to significantly increase [62]. Importantly, CAIX knockdown dramatically were found to reduce hypoxia- or high-glucose-induced invasive abilities [62]. Our results support the findings of these studies in that GBM had higher CAIX expression in response to hypoxia, leading to cell migration, and the inhibition of CAIX significantly reduced GBM migration and monocyte adhesion. Our findings also support previous reports that the knockdown of CAIX decreases GBM motility and monocyte adhesion.

Tumor and tumor-infiltrating cells are known to secrete various molecules, such as angiogenic IL-8, immunomodulatory IL-10, and TGF-β, all of which have both angiogenic and immunomodulatory functions [63]. Glioma-derived CSF-1, also known as macrophage colony-stimulating factor, induces a shift of microglia/macrophages toward M2-type TAMs, which enhance tumor growth. Moreover, from low-grade glioma to GBM tumors contain increasing numbers of macrophages, which represent the M2-type markers CD163 and CD204 [64]. TAMs can be acutely targeted via the inhibition of CSF-1 or the CSF-1 receptor (CSF-1R). Additionally, CSF-1 inhibitors are currently being evaluated in clinical trials [65], of which the most effective is the small-molecule CSF-1R inhibitor PLX3397 [66]. It has been reported that monocytes/macrophages are stimulated to express PD-L1 in response to IL-10 produced by GBM, resulting in upregulated PD-L1 expression and the immunosuppressive M2 phenotype [67]. Importantly, blocking the PD-1/PD-L1 pathway by antibodies was found to achieve clinical cure in advanced brain metastasis [68]. Clinical case reports also showed that PD-1 antibody therapy has evident therapeutic effects in GBM patients [69]. The present study supports the findings of these previous studies in that the higher Arg1 and IL-10 levels were expressed in co-cultured monocytes with GBM, and the higher PD-L1 levels were expressed in GBM in the co-cultured system. Additionally, CAIX and immunomodulatory molecules PD-L1, TGF-β, and CSF-1 were highly expressed in the tumor of the GBM-bearing mice brains.

It has been reported that the amplification or mutation of EGF receptors is observed in 50% of GBMs [50,70], which are associated with poor patient outcomes and survival rates [71]. On the contrary, the high expression of STAT3 in glioma is associated with a poor prognosis [72] Phosphorylated STAT3 expression in human gliomas is associated with the pathologic grades and the degree of immune infiltration [73]. Our previous finding suggested that EGFR activation is associated with the activation of STAT3 signaling in the stimulation of an inflammatory response [51]. Furthermore, our previous study showed that STAT3 signaling is involved in the expression of EGFR-associated adhesion molecules and monocyte adhesion in GBM [52]. It has been demonstrated that the over-activation of STAT3 results in tumor invasiveness by matrix metallopeptidase secretion and the upregulation of the focal adhesion kinase (FAK) [74]. Our previous study also found that FAK/STAT3 signaling is involved in GBM migration and IL-8 production [45]. The initiation of EGFR signaling by EGF or constitutively active gene mutation (EGFRvIII) results in the activation of the subsequent upregulation of HIF-1α [75,76]. It has also been reported that STAT3 knockdown reduces HIF-1α mRNA and protein expression in hypoxia. Similar reports have also indicated that STAT3 increases HIF-1α protein stability and interacts with HIF-1α to bind to HIF-1 target gene promoters [77]. It has been demonstrated that a causal relationship exists between STAT3 activation and HIF-1α-mediated VEGF expression. It has been demonstrated that CAIX expression in tumor cells is regulated by HIF-1α transcription factor under hypoxic conditions [59]. The inhibition of HIF-1α expression reduces the induction of CAIX levels under hypoxic conditions in thyroid carcinoma [78]. Importantly, accumulating evidence has shown that STAT3 functions cooperatively with HIF-1α to transactivate HIF-1α target genes, such as *VEGF* and *CAIX* [79]. Other reports have also shown that the activation of STAT3 is involved in the regulation of CAIX expression [80,81]. Moreover, it has been reported that CAIX is required to maintain the stemness within the hypoxic region in breast tumors [54]. Some reports have demonstrated that STAT3 inhibition decreases both radiation resistance and stem cell numbers in pancreatic cancer [82]. The present study also revealed a higher STAT3 activation in response to hypoxia, and the inhibition of STAT3 decreased HIF-1α downstream target CAIX expression under hypoxic conditions.

DDW has been recognized as a promising new anticancer agent that inhibits human lung carcinoma cell growth [83]. Defective mitochondria in renal cell carcinoma can impose tumor transformation by deuterium, and depletion of deuterium may act as a metabolic therapeutic adjuvant. Additionally, DDW production is the mitochondrial downstream mechanism of targeted anticancer drugs such as Avastin^®^ [84] and Gleevec^®^ [85]. Moreover, CAIX knockdown enhances the effect of Avastin^®^ treatment, reducing colon tumor growth [29]. However, the DDW yield of ketogenic substrates may be limited in primary brain tumors, and experimental xenografts and DDW for cytoplasmic hydrogen exchange reactions need to be incorporated into dieting protocols to achieve more favorable clinical outcomes [86].

## 4. Materials and Methods

### 4.1. Materials

The HIF-1α inhibitor (400083) was purchased from Calbiochem (San Diego, CA, USA). U104, DMOG, and AG1478 were purchased from Sigma-Aldrich (St. Louis, MO, USA). Stattic, erlotinib, and gefitinib were purchased from Selleckchem (Houston, TX, USA). Primary antibodies specific for HIF-1α (610958) were purchased from BD Biosciences (San Jose, CA, USA). Primary antibodies specific for HIF-1α (NB100-479) and CAIX (NB100-417) were purchased from Novus Biologicals (Centennial, CO, USA). Primary antibodies specific for glyceraldehyde 3 phosphate dehydrogenase (SI-G8795) and α-tubulin (SI-T5168) were purchased from Sigma-Aldrich. Primary antibodies specific for p300 (sc-584), proliferating cell nuclear antigen (sc-56), β-actin (sc-47778), and immunoglobulin G (IgG) control were purchased from Santa Cruz Biotechnology (Santa Cruz, CA, USA). Primary antibodies specific for p-STAT3 (Tyr705, 9131), Ac-STAT3 (Lys685, 2523), EGFR (4267), p-EGFR (Tyr1045, 2237), and p-EGFR (Tyr 1068, 2220) were purchased from Cell Signaling Technology (Danvers, MA, USA). On-Target SMART pool p300 siRNA and control non-targeting siRNA were purchased from Dharmacon (Lafayette, CO, USA). Small hairpin RNAs of CAIX and control were obtained from the National Center for Genome Medicine (Taipei, Taiwan).

### 4.2. Plasmid Constructs

The constructs used were as previously described [52]. In brief, the promoter region of the *CAIX* gene (−939 to −1 bp) was amplified by PCR, and the fragment was ligated into the luciferase reporter plasmid pGL4.17 (Promega, Madison, WI, USA) to generate the wild-type construct. The overexpressing constructs containing the coding sequence (CDS) of *CAIX* (1380 base pair, 33-1412) were amplified by the PCR of cDNA obtained from U251 cells. PCR products were cloned into a pcDNA3.1 vector (Invitrogen, Carlsbad, CA, USA) between the XhoI and HindIII restriction sites. All constructs were sequenced to verify that they contained CDS inserts.

### 4.3. Animals

Seven-week-old C57BL/6 male mice were obtained from the National Laboratory Animal Center (Taipei, Taiwan). Mice were housed under standard laboratory conditions (21 ± 2 °C, a 12 h light/dark cycle, and with food and water available ad libitum). All animal procedures were performed in accordance with the Animal Care and Use Guidelines of China Medical University (China Medical University, Taichung, Taiwan). Intracranial injections were administered according to protocols approved by the China Medical University Animal Care and Use Committee (Animal Study Proof No.: CMUIACUC-2019-086. 2019-2022).

### 4.4. Cell Culture

U87 human GBM, ALTS1C1 mouse GBM, and THP-1 human monocytes were obtained from the Bioresource Collection and Research Center (BCRC Nos. 60360, 60582, and 60430, Taoyuan, Taiwan). U251 human GBM was obtained from the Japanese Collection of Research Bioresources (JCRB) Cell Bank (JCRB NO. IFO50288, Japan). U251-GFP (green fluorescent protein) and U87-GFP cells were established and maintained in our laboratory. Briefly, pAS2-EGFP.puro plasmids were transfected into either U251 or U87 cells. Stable clones were initially selected using 1.5–2.5 μg/mL puromycin (Sigma) for 2 weeks and subsequently maintained in 2 μg/mL puromycin in a culture medium. U251-GFP or U87-GFP cells were seeded at 100 cells into 96 well plates and grown for 2 weeks. Multiple clones of U251-GFP and U87-GFP were established to stably express GFP, and their expressions were verified using fluorescence microscopy and Western blotting [52]. U87 and U251 were maintained in Minimum Essential Medium, ALTS1C1 was maintained in Dulbecco’s Modified Eagle Medium, and THP-1 was maintained in RPMI-1640. All cells were cultured in a medium supplemented with 10% fetal bovine serum, 100 U/mL penicillin, and 100 mg/mL streptomycin, and they were incubated at 37 °C in a humidified atmosphere containing 5% CO_2_ and 95% air. All cells used in this study were tested for mycoplasma. For hypoxic exposure, cells were maintained at 37 °C in a sealed acrylic chamber flushed with a gas mixture containing 1% O_2_, 5% CO_2_, and 94% N_2_.

### 4.5. Intracranial Tumor Implantation

ALTS1C1 was freshly prepared and adjusted to 5 × 10^7^ cells/mL before implantation. Intracranial injections were administered according to protocols approved by the China Medical University Animal Care and Use Committee. Briefly, 8-week-old C57BL/6 male mice (four mice) were anesthetized with isoflurane and placed in a stereotactic frame, and the skulls were exposed by incision (China Medical University, Taichung, Taiwan). In each skull, a hole was made 0.8 mm anterior at 2.5 mm to the right of the bregma, and cells (2 μL) were injected using a 10-μL Hamilton syringe with a 26S-gauge needle mounted in a stereotactic holder. The syringe was lowered to a depth of 3 mm, and the cells were injected at a rate of 0.4 μL/min. After intracranial implantation, a 5-min waiting period was observed before slowly withdrawing the syringe to prevent reflux. The skull was subsequently cleaned, and the incision was sutured. Tumors were allowed to grow till the indicated periods and then sacrificed. At the end of the experiment, the mice were sacrificed following CO_2_ asphyxiation under isoflurane anesthesia, and tumor tissues were obtained for real-time PCR assay.

### 4.6. Analysis of Gene Expression Omnibus (GEO) Glioma Microarray Datasets

In this study, already available DNA microarray data were obtained from NCBI GEO (accession number: GSE4290), and gene expression was reanalyzed using the GraphPad Prism 6 software. The study cohort comprised 180 patients from the Henry Ford Hospital with histologically confirmed gliomas of different grades as follows: non-tumors (23), grade II (45) (astrocytomas [7] and oligodendrogliomas [38]), grade III (31) (astrocytomas [19], oligodendrogliomas [12]), and grade IV astrocytomas (81) (GBM) [87]. The *HIF-1*α, *CAIX*, and *NHE1* gene expression values were obtained from the GSE4290 dataset to evaluate the correlations with the grading of human gliomas.

### 4.7. Western Blotting

The procedure of Western blotting was described previously [88]. Briefly, cell lysates were separated using sodium dodecyl sulfate-polyacrylamide gel electrophoresis and transfected onto polyvinylidene difluoride membranes (Millipore, Bedford, MA, USA). The membranes were blocked with 5% nonfat dry milk in tris-buffered saline and probed overnight with primary antibodies at 4 °C, followed by incubation with peroxidase-conjugated secondary antibodies (Santa Cruz Biotechnology). The blots were visualized using enhanced chemiluminescence and Kodak X-OMAST LS film (Eastman Kodak, Rochester, NY, USA). Quantitative data were obtained using ImageJ software.

### 4.8. Cell Transfection

GBM cells were transiently transfected with wild-type CAIX, CAIX luciferase reporter plasmids, the shRNA of CAIX, or the DN mutant of HIF-1α using Lipofectamine (LF)3000 (Invitrogen) for 24 h. Plasmid DNA and LF3000 were premixed in serum-free medium for 5 min and subsequently applied to the cells. The LF3000-containing medium was replaced with a fresh serum-free medium after 24 h. GBM cells were transiently transfected with SMART pool siRNA against p300 or control siRNA (Dharmacon, Lafayette, CO, USA) using DharmaFECT transfection reagents (Dharmacon). Either the siRNA or the negative control was premixed with the DharmaFECT transfection reagent in a serum-free medium for 25 min and was subsequently used to transfect the cells. After 24 h, the reagent-containing medium was replaced with fresh serum-free medium.

### 4.9. RNA Extraction and Quantitative Real-Time Polymerase Chain Reaction

Total RNA was extracted from cells using a TRIzol reagent (Invitrogen) and quantified using a BioDrop spectrophotometer (Cambridge, UK). Target mRNA levels were detected using quantitative real-time PCR. The reverse transcription (RT) reaction was performed using 2 μg of total RNA converted into cDNA using the Invitrogen RT Kit and amplified using the following oligonucleotide primers: hHIF-1α, 5′-TTACA GCAGC CAGAC GATCA-3′ and 5′-CCCTG CAGTA GGTTT CTGCT-3′; hCAIX, 5′-ACCCT CTCTG ACACC CTGTG-3′ and 5′-GGCTG GCTTC TCACA TTCTC-3′; hMCT4, 5′-GAGTT TGGGA TCGGC TACAG-3′ and 5′-CGGTT CACGC ACACA CTG-3′; hNHE1, 5′-GTTGG GTTTG GCACT CAACT-3′ and 5′-GAAGA CAGGG CTACC TGCTG-3′; GLUT1, 5′-CTGCT CATCA ACCGC AAC-3′ and 5′-CTTCT TCTCC CGCAT CATCT-3′; GLUT3, 5′-ACCGG CTTCC TCATT ACCTT-3′ and 5′-AGGCT CGATG CTGTT CATCT-3′; h36B4, 5′-AGATG CAGCA GATCC GCAT-3′ and 5′-GTTCT TGCCC ATCAG CACC-3′; mPD-L1, 5′-GCTCC AAAGG ACTTG TACGT G-3′ and 5′-TGATC TGAAG GGCAG CATTT C-3′; mCAIX, 5′-TGCTC CAAGT GTCTG CTCAG-3′ and 5′-CAGGT GCATC CTCTT CACTG G-3′; mTGF-β, 5′-CTCCC GTGGC TTCTA GTGC-3′ and 5′-GCCTT AGTTT GGACA GGATC TG-3′; mCSF-1, 5′-GGCTT GGCTT GGGAT GATTC T-3′ and 5′-GAGGG TCTGG CAGGT ACTC-3′; and m36B4, 5′-AGATG CAGCA GATCC GCAT-3′, and 5′-GTTCT TGCCC ATCAG CACC-3′. The qPCR protocol was described in our previous report [89]. The qPCR was performed with the StepOne Plus System (Applied Biosystems) using SYBR Green Master Mix (Applied Biosystems, Foster City, CA, USA). The PCR was performed as follows: 45 cycles of 95 °C for 1 s and 60 °C for 20 s. The expression level of 36B4 was used as an internal control to normalize the expression levels of the target mRNAs. The threshold was set within the linear phase of target gene amplification to calculate the cycle number at which the transcript was detected (denoted as CT).

### 4.10. Monocyte-Binding Assay

The procedure of the monocyte-binding assay was described previously [52]. Briefly, THP-1 cells were labeled with 0.1 μg/mL of BCECF/AM (Invitrogen, Green) at 37 °C for 1 h, followed by washing twice with a growth medium. GBM cells were treated with U104 or transfected with wild-type CAIX. The medium was removed from the wells, and 2.5 × 105 BCECF/AM-labeled-THP-1 cells were added to monolayer GBM cells. After incubation at 37 °C for 30 min, the wells were gently washed twice with a warm growth medium to remove non-adherent cells. The cells were subsequently photographed under a fluorescence microscope to calculate the adherent cells (count and average the total number of cells in 5 random fields at 100× total magnification).

### 4.11. Cell Migration Assay

The cell migration assay was performed using Costar Transwell inserts (8-μm pore size, Costar, NY, USA) in 24-well plates, as previously described [90]. Prior to the migration assay, the GBM cells were treated with U104, an HIF-1α inhibitor, or transfected with wild-type CAIX. Approximately 1.5 × 104 GBM cells in 200 μL of serum-free medium were placed in the upper chamber, and 400 μL of the same medium was placed in the lower chamber. The plates were incubated for 24 h at 37 °C under hypoxic conditions (1% O_2_). The cells that migrated through the filters were fixed with 3.7% formaldehyde for 5 min and stained with 0.05% crystal violet for 30 min. Cells on the upper side of the filters were wiped with cotton-tipped swabs, and the cells on the underside of the filter were photographed using a digital camera mounted on to a microscope (count and average the total number of cells in 5 random fields at 100× total magnification).

### 4.12. GBM-Monocyte Co-Cultured System

For co-cultures, cultures of GBM cells including U87-GFP or U251-GFP were seeded at 2 × 10^6^ cells per 100-mm dish and maintained in a normoxic condition for 6 h. Subsequently, incubation with 2 × 10^6^ THP-1 cells per dish was performed, which was maintained in a hypoxic condition. After 48 h, co-cultured cells were isolated from single cells in a culture medium. After cell separation, cells were washed twice with a phosphate-buffered saline (PBS), and flow cytometry analysis was performed.

### 4.13. Flow Cytometry Analysis

For co-culture, flow cytometric analysis was performed using a BD Cytofix/Cytoperm Plus fixation/permeabilization protocol (BD Biosciences, San Jose, CA, USA), as previously described [52]. The co-cultured cells were blocked for nonspecific binding using anti-CD16/CD32 antibodies (BioLegend, San Diego, CA, USA, 101301) for 20 min, and the co-cultured cells were fixed and permeabilized with BD Cytofix/Cytoperm solution for 20 min at 4 °C. The co-cultured cells were washed with a BD Perm/Wash buffer, resuspended in a BD Perm/Wash buffer, and incubated with anti-CD206-PE (eBioscience, 12-2069), anti-ARG1-PE (BioLegend, 369704), anti-IL-10-eFluor 660 (eBioscience, 50-7108), anti-IFN-γ-PE (eBioscience, 12-7319), anti-TNF-α-APC (eBioscience, 17-7349), or anti-PD-L1-APC (BioLegend, 329707) for 30 min at 4 °C. The co-cultured cells were subsequently washed with a Perm/Wash buffer and resuspended in a Flow Cytometry Staining Buffer.

### 4.14. Luciferase Reporter Assay

GBM cells were transfected with CAIX firefly luciferase reporter plasmids and SV40-renilla luciferase control plasmids using LF3000 (Invitrogen) according to the manufacturer’s recommendations. At 24 h after transfection, the cells were pretreated with inhibitors for 30 min and subsequently exposed to hypoxic conditions for 24 h. Cell extracts were subsequently prepared, and firefly and renilla luciferase activities were measured.

### 4.15. Preparation of Cytosolic and Nuclear Extracts

Nuclear extracts were prepared as previously described [91]. Briefly, cells were rinsed with cold PBS and resuspended in a hypotonic buffer (10 mM HEPES, pH 7.6, 10 mM KCl, 1 mM dithiothreitol, 0.1 mM ethylenediaminetetraacetic acid (EDTA), and a protease inhibitor cocktail) for 10 min on ice. The cytosolic proteins were separated by centrifugation at 10,000× *g* for 2 min. The supernatants containing the cytosolic proteins were collected, and the pellets containing the nuclear fraction were resuspended in a buffer (20 mM HEPES, pH 7.6, 1 mM EDTA, 1 mM dithiothreitol, 0.4 M sodium chloride, 25% glycerol, and a protease inhibitor cocktail) for 30 min on ice. The suspensions were centrifuged again at 13,000× *g* for 20 min, and the supernatants containing the nuclear proteins were collected and stored at −80 °C.

### 4.16. Co-Immunoprecipitation (Co-IP) Assay

Co-IP assays were prepared as previously described [45]. Briefly, co-IP was performed with whole cell lysates (1 mg) incubated with p300 or non-specific IgG (2 μg) antibodies using protein G Mag Sepharose Xtra (GE Healthcare, Uppsala, Sweden) and a MagJET Separation Rack (Thermo Scientific, Hudson, NH, USA), according to the manufacturer’s instructions.

### 4.17. Statistical Analyses

The statistical analysis was performed using GraphPad Prism 8 software (GraphPad Software Inc., San Diego, CA, USA). Data are presented as the mean ± standard error, and all experiments were performed with three biologically independent replicates. Sample sizes were calculated to determine whether significant results were achieved. Statistical analyses between two samples were performed using Student’s *t*-test. No pretest was used to determine the sample size. No data points were excluded. Pearson’s correlation test was used to examine the association between HIF-1α and CAIX expression in patients with glioma. In all cases, a *p*-value < 0.05 was considered statistically significant. Subsequently, *n* and *p*-values are indicated in the figure legends.

## 5. Conclusions

In conclusion, we provided evidence that hypoxia induces HIF-1α-associated CAIX and pH-regulating protein expression through EGFR/STAT3 signaling in GBM. Moreover, CAIX is a critical modulator that regulates GBM migration and monocyte adhesion. The results of co-cultured system indicated theenhancement of the M2 marker, CD206, Arg1, and IL-10 in monocytes, as well as the subsequent upregulation of PD-L1 in GBM under hypoxic conditions, thus consequently promoting a suppressive immune microenvironment and enhancing GBM progression. Our results provide a new perspective in assessing the development of GBM and provide novel insights into the tumor microenvironment, possibly identifying new treatment strategies for cancer diagnosis and therapy.

## Supplementary Materials

The following are available online at https://www.mdpi.com/1422-0067/21/16/5838/s1, Figure S1: Hypoxia induces CAIX and pH-regulating proteins expression in GBM. Figure S2: Hypoxia induces HIF-1α and CAIX expression in GBM. Figure S3: Overexpression of CAIX in human GBM cells. Figure S4: Expression of PD-L1 on THP-1 monocytes under hypoxic conditions.
