# Supplementary material for: CAIX Regulates GBM Motility and TAM Adhesion and Polarization through EGFR/STAT3 under Hypoxic Conditions
Source: Int J Mol Sci. 2020 Aug 14;21(16):5838. doi: 10.3390/ijms21165838 (PMC7461579; doi:10.3390/ijms21165838)
Supplement: Supplementary file 1 [file ijms-21-05838-s001.pdf]

Supplementary data

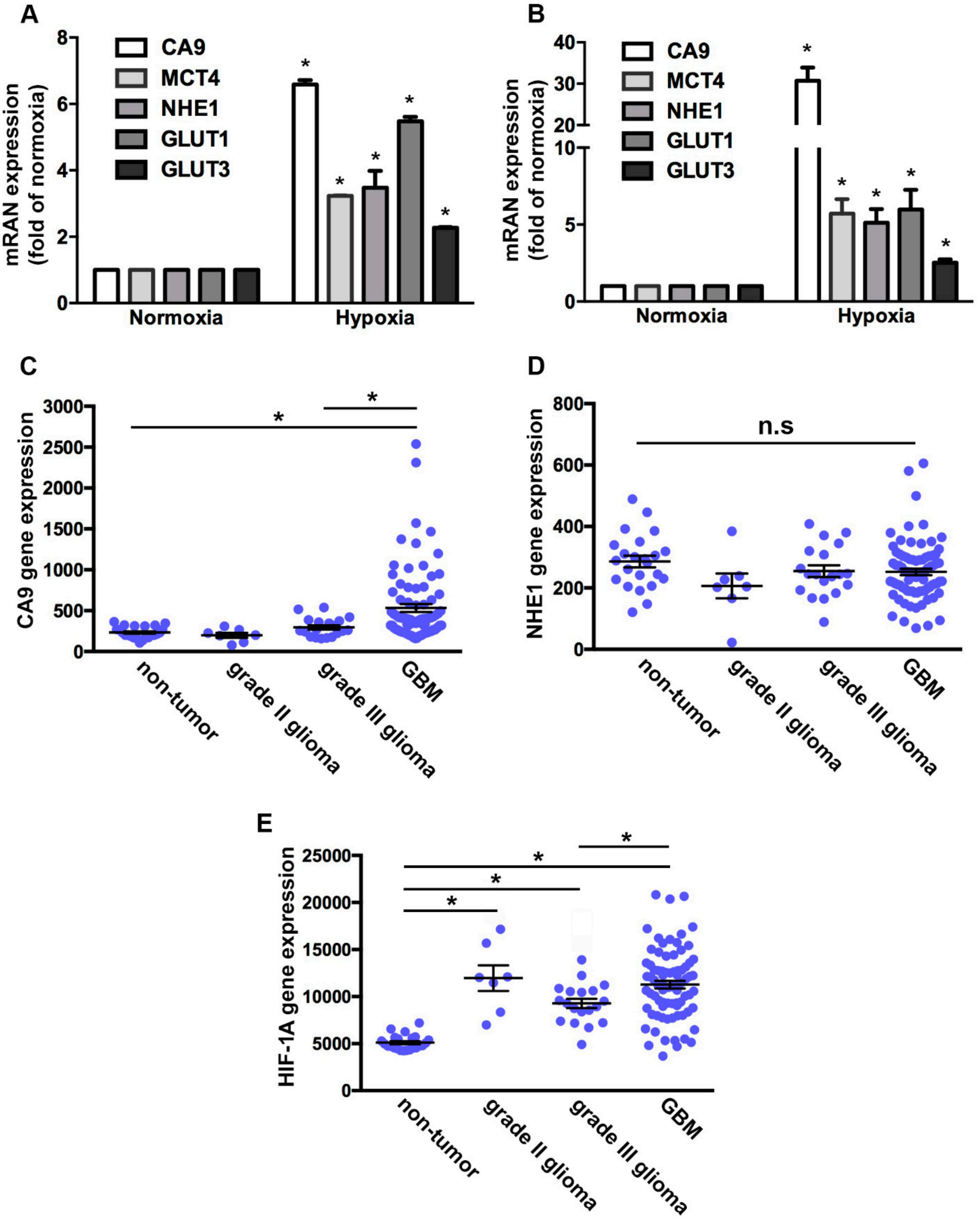

Figure S1. Hypoxia induces CAIX and pH-regulating proteins expression in GBM. U87 were exposed to hypoxic conditions (1% O_2) for 6 h (**A**) or 24 h (**B**), mRNA expressions were analyzed by qPCR for indicated proteins. *$p < 0.05$ compared with the normoxia group. One-way ANOVA with a post-hoc Bonferroni test was used to examine the significance of the mean. Quantitative data are presented as mean ± s.e.m. (representative of n = 3). Messenger RNA levels of CAIX (**C**), NHE (**D**), and HIF-1A (**E**) in patients' specimens from

human glioma microarray datasets GSE4290. *$p < 0.05$ compared with the non-tumor or grade III glioma. ns: not significant. One-way ANOVA with a post-hoc Bonferroni test was used to examine the significance of the mean.

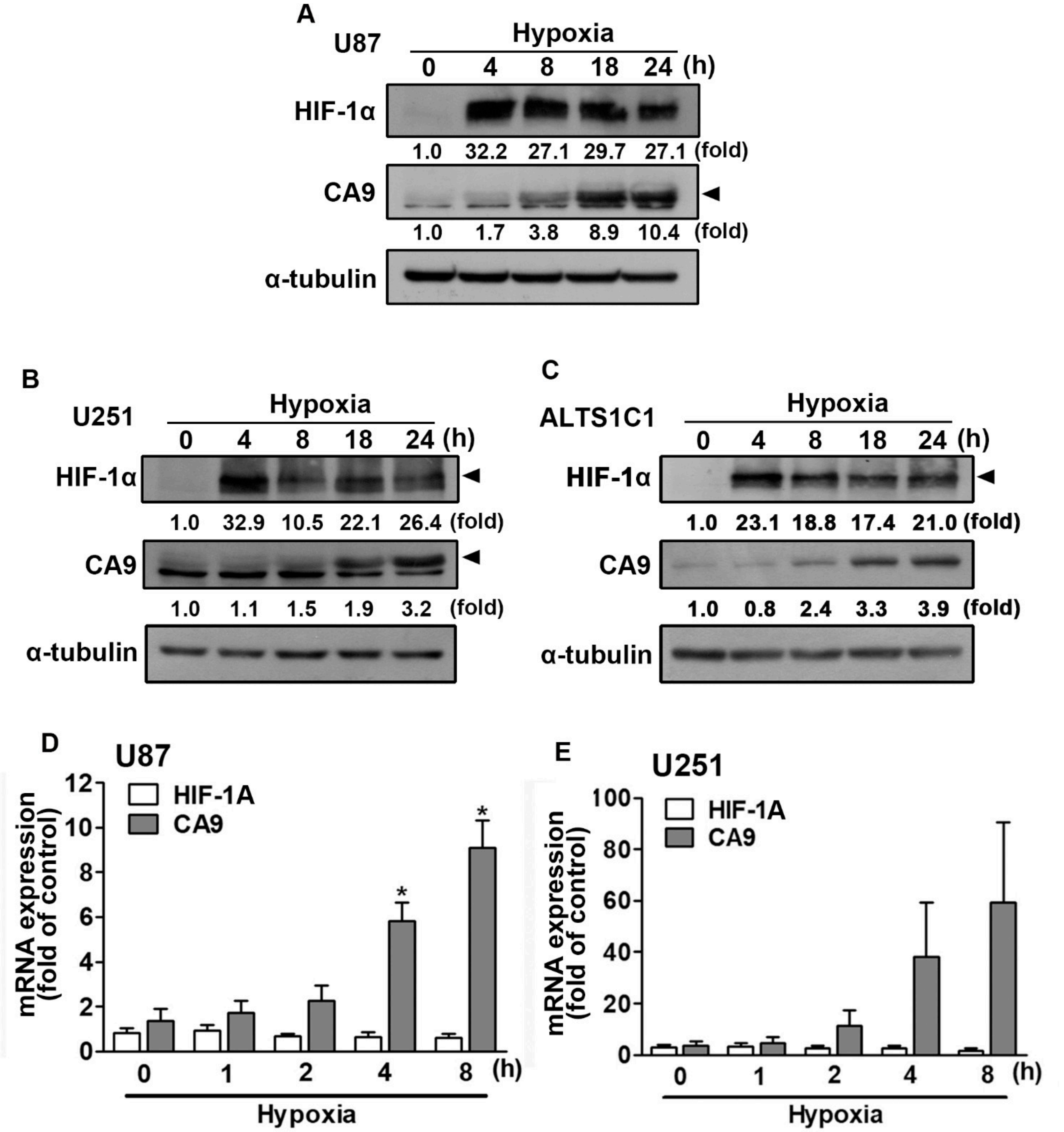

Figure S2. Hypoxia induces HIF-1α and CAIX expression in GBM. U87 (**A**), U251 (**B**), and ALTS1C1 (**C**) were exposed to hypoxic conditions (1% O_2) for indicated time periods (4, 8, 16, or 24 h). HIF-1α and CAIX protein expression were determined by western blotting using whole cell lysates. U87 (**D**) and U251 (**E**) were exposed to hypoxia condition (1% O_2) for indicated time periods (1, 2, 4, or 8 h), and *HIF-1A* and *CAIX* mRNA expression were determined using qPCR. Each time points was compared with the same time periods of normoxic conditions. *p < 0.05 compared with the control group. Quantitative data are presented as mean ± s.e.m. (representative of n = 3).

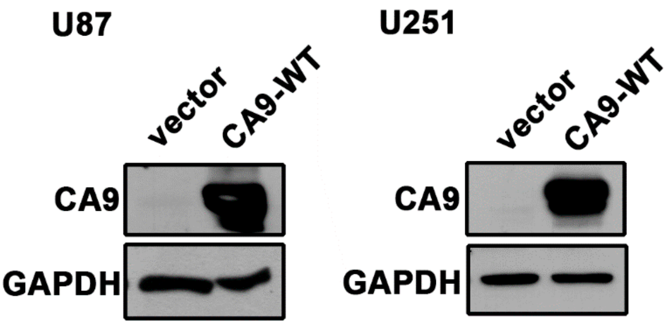

Figure S3. Overexpression of CAIX in human GBM cells. U87 and U251 cells were transfected with empty vector or wild-type plasmid of CAIX for 24 h and subsequently exposed to hypoxic conditions (1 % O_2) for another 24 h. CAIX expression was determined by western blotting using whole cell lysates.

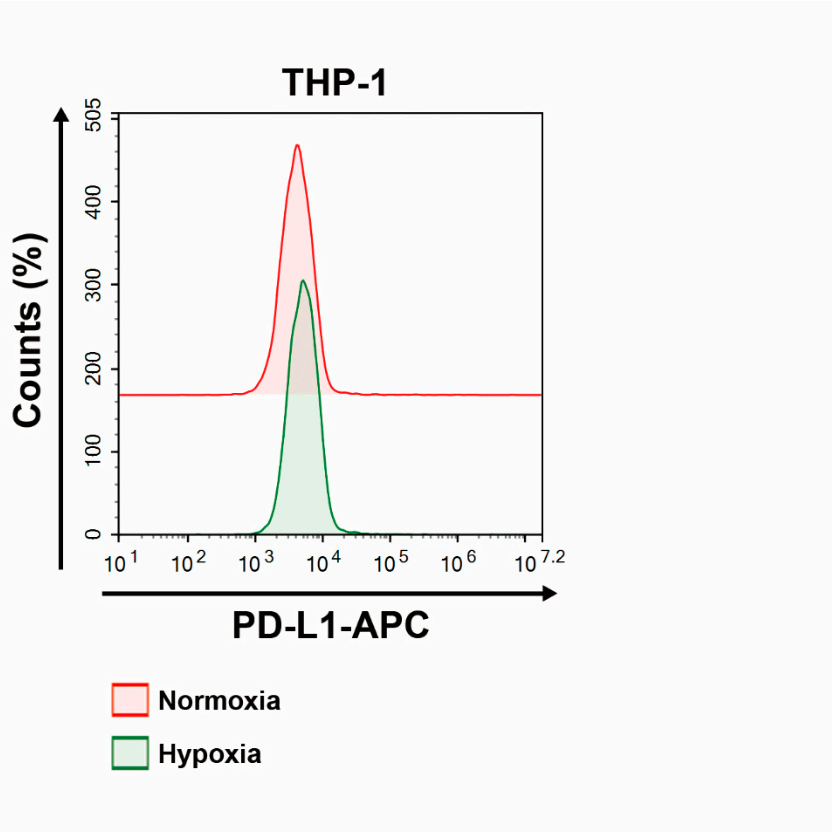

Figure S4. Expression of PD-L1 on THP-1 monocytes under hypoxic conditions. THP-1 monocytes were incubated under hypoxic or normoxic conditions for 48 h. The levels of programmed death ligand 1 (PD-L1) were determined by flow cytometry.
